# Exploring the Role of Drug Repurposing in Bridging the Hypoxia–Depression Connection

**DOI:** 10.3390/membranes13090800

**Published:** 2023-09-17

**Authors:** Ana Salomé Correia, Lara Marques, Armando Cardoso, Nuno Vale

**Affiliations:** 1 OncoPharma Research Group, Center for Health Technology and Services Research (CINTESIS), Rua Doutor Plácido da Costa, 4200-450 Porto, Portugal; anncorr07@gmail.com (A.S.C.); lara.marques2010@hotmail.com (L.M.); 2 CINTESIS@RISE, Faculty of Medicine, University of Porto, Alameda Professor Hernâni Monteiro, 4200-319 Porto, Portugal; cardosoa@med.up.pt; 3 Institute of Biomedical Sciences Abel Salazar (ICBAS), University of Porto, Rua de Jorge Viterbo Ferreira 228, 4050-313 Porto, Portugal; 4 Department of Community Medicine, Information and Health Decision Sciences (MEDCIDS), Faculty of Medicine, University of Porto, Rua Doutor Plácido da Costa, 4200-450 Porto, Portugal; 5 NeuroGen Research Group, Center for Health Technology and Services Research (CINTESIS), Rua Doutor Plácido da Costa, 4200-450 Porto, Portugal; 6 Unit of Anatomy, Department of Biomedicine, Faculty of Medicine, University of Porto, Alameda Professor Hernâni Monteiro, 4200-319 Porto, Portugal

**Keywords:** drug repurposing, hypoxia-inducible factor-1, serotonin receptors, cobalt chloride, Echinomycin

## Abstract

High levels of oxidative stress are implicated in hypoxia, a physiological response to low levels of oxygen. Evidence supports a connection between this response and depression. Previous studies indicate that tryptophan hydroxylase can be negatively affected in hypoxia, impairing serotonin synthesis and downstream pathways. Some studies also hypothesize that increasing hypoxia-inducible factor-1 (HIF-1) levels may be a new therapeutic modality for depression. Hence, this study delved into the influence of hypoxia on the cellular response to drugs designed to act in depression. By the induction of hypoxia in SH-SY5Y cells through a hypoxia incubator chamber or Cobalt Chloride treatment, the effect of Mirtazapine, an antidepressant, and other drugs that interact with serotonin receptors (TCB-2, Dextromethorphan, Ketamine, Quetiapine, Scopolamine, Celecoxib, and Lamotrigine) on SH-SY5Y cellular viability and morphology was explored. The selection of drugs was initially conducted by literature search, focusing on compounds with established potential for employment in depression therapy. Subsequently, we employed in silico approaches to forecast their ability to traverse the blood–brain barrier (BBB). This step was particularly pertinent as we aimed to assess their viability for inducing potential antidepressant effects. The effect of these drugs in hypoxia under the inhibition of HIF-1 by Echinomycin was also tested. Our results revealed that all the potential repurposed drugs promoted cell viability, especially when hypoxia was chemically induced. When combined with Echinomycin, all drugs decreased cellular viability, possibly by the inability to interact with HIF-1.

## 1. Introduction

Oxygen is essential for the normal functioning of the human body. Indeed, decreased levels of oxygen (hypoxia) lead to high levels of stress, culminating in processes such as inflammation, brain injury, cardiovascular defects, mitochondrial oxidative stress, and apoptosis, failing in the maintenance of homeostasis. Inadequate oxygen delivery to the tissues, low blood supply, or low levels of oxygen in the blood are usually the basis of this process [1,2,3,4]. Hypoxia is also implicated in anxiety and depressive disorders. Studies indicate that hypoxia can disrupt the normal neurohormonal balance in the brain, increasing the potential for depressive symptoms [5]. Indeed, in people that live at high altitudes and are chronically exposed to hypoxia, increased suicide rates are observed, possibly connected to the hypoxic exposure. Indeed, increased altitude can contribute to the reduced partial pressure of arterial oxygen (PaO_2_) and, because the brain partial pressure of oxygen is limited by this parameter, to reduced brain oxygen, a risk factor for mood disorders [6,7,8]. It is also suggested that in situations of hypoxia, the enzyme that produces serotonin (tryptophan hydroxylase) may not function normally due to lack of oxygen saturation, decreasing serotonin synthesis, which is connected with depressive disorders [7]. The dysfunction of the serotonin receptor 1A (5-HT1A) plays a key role in the development of depression [9]. A study that focused on the effects of hypoxia on this receptor revealed that after the exposure of cells to hypoxia, a significant reduction in ligand binding was observed, along with the disruption of downstream signaling of the 5-HT1A receptor, particularly cAMP-mediated signaling [10]. Nevertheless, besides the well-known role of serotonin receptors in depression, there are other important players, particularly neurotrophic and inflammatory factors, hypothalamic-pituitary-adrenal (HPA) axis, epigenetic modifications, oxidative stress, and imbalance in neurotransmitter levels besides serotonin, such as dopamine and glutamate. Thus, exploring hypoxia in the context of depression may involve different targets [11].

In response to hypoxia, there are adaptive responses. One of the most important regulators of this response is the major transcription factor denominated Hypoxia-inducible factor-1 (HIF-1). This dimeric complex presents an alpha subunit (HIF-1α), highly sensitive to oxygen and degraded by the proteasome in normoxic cells [12]. Under hypoxic conditions, HIF-1 targets genes especially related to angiogenesis, cell proliferation/survival, and glucose/iron metabolism, which are essential to cell survival in hypoxic environments [13]. Indeed, some studies hypothesize that increasing the levels of HIF-1 may be a new therapeutic alternative to depression by facilitating creatine metabolism in the brain [14]. Additionally, the administration of the potent HIF-1 inhibitor topotecan worsened the severity of the depressive-like symptoms in an experimental model of depression in rats [15]. Another study also revealed that FG-4592 (an inhibitor of prolyl hydroxylase) improved depressive-like symptoms by mediating neurogenesis and synaptic plasticity through HIF-1 [16].

The molecular and cellular characterization of the hypoxia response and pathways involved in this mechanism is greatly possible due to hypoxia models in cell culture [1]. Thus, our study aims to understand the effect of Mirtazapine, a classical antidepressant [17], and other drugs with the reported potential to be repurposed in the therapy of depression, namely TCB-2, Dextromethorphan, Ketamine, Quetiapine, Scopolamine, Celecoxib, and Lamotrigine [18,19,20], on human neuroblastoma SH-SY5Y cells under hypoxia and under inhibition of HIF-1 by Echinomycin, a potent HIF-1α inhibitor that binds to DNA by bifunctional intercalation, blocking the binding of HIF-1α [21]. In a preliminary phase, we conducted a screening of multiple potential drugs that interact mainly with serotonin receptors to identify potential candidates for repurposing in depression treatment. Considering the neurological nature of depression and our focus on evaluating the effect of these compounds on neuroblastoma cells, our drug selection criteria were centered on their ability to cross the blood–brain barrier (BBB), a highly selective and protective barrier that separates the bloodstream from the brain’s extracellular fluid and is important to control the entry of drugs into the brain. Thus, we identified TCB-2, Dextromethorphan, Ketamine, Quetiapine, Scopolamine, Celecoxib, and Lamotrigine as promising candidates for repurposing in the treatment of this disorder. Subsequently, their effects on SH-SY5Y cells under hypoxic conditions and in combination with Echinomycin were assessed. To induce hypoxia, we used a hypoxia incubator chamber and Cobalt Chloride (CoCl_2_), usually used as a hypoxia-mimetic agent that can block HIF-1α protein degradation under normoxic conditions and can circumvent a common problem faced with the use of the hypoxia chamber, particularly the exact regulation of oxygen levels. Indeed, the use of CoCl_2_ in vitro increases HIF-1α/2α in a dose- and time- dependent manner, similar to that observed in hypoxia [1,22].

Our main findings reveal that the above-mentioned drugs could induce cellular viability, especially when combined with CoCl2, possibly by inducing HIF-1-related adaptive pathways. Additionally, to explore the role of these drugs on HIF-1 signaling, we combined them with Echinomycin under a hypoxic environment induced by CoCl2, revealing that all drugs decreased cellular viability, possibly by the inability to interact with HIF-1 signaling, inhibited by Echinomycin. Thus, our study highlights the possible role of hypoxia in depression, which is a common and complex disorder, urgent to be deeply studied [11].

## 2. Materials and Methods

### 2.1. Materials

Dulbecco’s modified Eagle’s medium (DMEM; cat. no. FG0415) and fetal bovine serum (FBS; cat. no S0615) were obtained from Millipore Sigma (Merck KGaA, Darmstadt, Germany). Penicillin/streptomycin (cat. no. P4333), thiazolyl blue tetrazolium bromide (MTT; cat. no. M5655), Dextromethorphan hydrobromide (cat. no. D9684-5G), Scopolamine hydrobromide (cat. no. PHR1470-500MG), Celecoxib (cat. no. PZ0008-5MG), and Cobalt (II) Chloride hexahydrate (cat. no. 255599-5G) were obtained from Sigma-Aldrich (Merck KGaA, Darmstadt, Germany). Mirtazapine (cat. no. 19994), Quetiapine hemifumarate (cat. no. 14152), and Lamotrigine (cat. no 14428) were purchased from Cayman Chemical Company (Ann Arbor, MI, USA). TCB-2 (cat. no. 2592) was purchased from Tocris (Bristol, UK), and Ketamine Hydrochloride (cat. no. DRE-C14531000) was obtained from LGC Standards (Middlesex, UK). The hypoxia incubator chamber was obtained from StemCell (Vancouver, British Columbia, Canada; cat no. 27310).

### 2.2. Preliminary Screening of Drugs with Potential for Repurposing in the Treatment of Depression

To identify the best drug candidates for this study, several compounds with documented potential to be repurposed in depression treatment were screened using ADMET Predictor^®^ (Version 10.4; Simulation Plus Inc., Lancaster, CA, USA). The selection of compounds for our study was predicated upon their capacity to cross the blood–brain barrier (BBB), as we intended to evaluate their potential antidepressant effect in neuroblastoma cells.

The chemical structure of each drug was generated in MedChem Designer (Version 5.5.; Simulation Plus Inc., Lancaster, CA, USA) through SMILES input and subsequently imported into ADMET Predictor^®^ as MOL files. All drugs were characterized according to their physicochemical and pharmacokinetic properties. This analysis encompassed lipophilicity parameters, molecular weight, solubility, BBB permeability, and other drug’s attributes estimated by this software tool.

### 2.3. Cell Culture

SH-SY5Y cell line was purchased from American Type Culture Collection (Manassas VA, USA). Cells were incubated at 37 °C (5% CO_2_) and cultured in DMEM, 10% FBS, and 1% penicillin (1000 U/mL)/streptomycin (10 mg/mL), according to recommendations. Before each new assay, cells were trypsinized (0.25% trypsin-EDTA) and centrifuged. The seeding was conducted with a density of 1.0 × 10^5^ cells/mL in 96-well plates (200 μL/well).

### 2.4. Cell Treatments

Mirtazapine was prepared as previously described [23]. All the compounds were dissolved in DMSO (0.1% in cell culture medium), except Scopolamine (sterilized water 0.1% in cell culture medium) and CoCl_2_ (sterilized water 1% in cell culture medium). For Mirtazapine, TCB-2, Dextromethorphan, Ketamine, Quetiapine, Scopolamine, Celecoxib, and Lamotrigine, concentrations of 10 nM and 20 µM were tested. For CoCl_2_ and Echinomycin, concentrations tested ranged 0.1–1 mM and 0.1–5 nM, respectively. All the treatments were tested for a period of 48 h. Vehicles were composed of DMSO 0.1% or sterilized water 1%. For double or triple combinations, they were composed of, respectively, DMSO 0.2%/sterilized water 2% and DMSO 0.3%/sterilized water 3%.

### 2.5. Cell Morphology Visualization

Cells were observed and photographed after each treatment (48 h) using Leica DMI6000 B Automated Microscope (Leica, Wetzlar, Germany).

### 2.6. MTT Assay

After exposure to the different treatments for 48 h, cellular viability was evaluated by thiazolyl blue tetrazolium bromide (MTT) assay. First, the culture medium was removed, and then MTT (0.5 mg/mL in PBS) was added to each plate well (100 µL/well) following a 3h period of incubation at 37 °C, protected from the light. Finally, MTT was removed and DMSO (100 µL/well) was added to the cells to dissolve the formed crystals. Then, 570 nm absorbance values were obtained from the automated microplate reader (Tecan Infinite M200, Zurich, Switzerland).

### 2.7. Hypoxia Models

For the induction of hypoxia, we used CoCl_2_ or the hypoxia chamber, as described in the introduction. For hypoxia induced with the chamber, each plate was placed in the hypoxia incubator chamber with a 2% O_2_, 10% CO_2_, and 88% N_2_ atmosphere. For hypoxia induced with CoCl_2_, we applied to the cells 0.1 mM CoCl_2_ for 48 h.

### 2.8. Statistical and Data Analyses

The results were expressed as mean ± SEM of three independent experiments. Statistical analyses between each vehicle and treatments and between treatments were carried out with one-away ANOVA followed by Dunnett’s or Tukey’s multiple comparisons test, respectively. Statistical significance was considered when *p* < 0.05. Statistical analyses and graphical construction were carried out using the software GraphPad Prism 8.02 (San Diego, CA, USA).

## 3. Results

### 3.1. Drugs with Potential for Repurposing in Depression Treatment

Several drugs were analyzed for inclusion in our study (Table 1) using ADMET predictor^®^. Indeed, the selection of drugs was initially guided by literature search, focusing on compounds with established potential for employment in depression therapy. Then, the selection criteria for the compounds were centered on their ability to cross the BBB. According to our predictions, Mirtazapine, TCB-2, Dextromethorphan, Ketamine, Quetiapine, Scopolamine, Celecoxib, and Lamotrigine demonstrated a high capacity to cross BBB. Therefore, these compounds were selected to proceed with our study.

### 3.2. The Effect of the Hypoxia Incubator Chamber on SH-SY5Y Cellular Viability and Morphology

**Figure 1:**
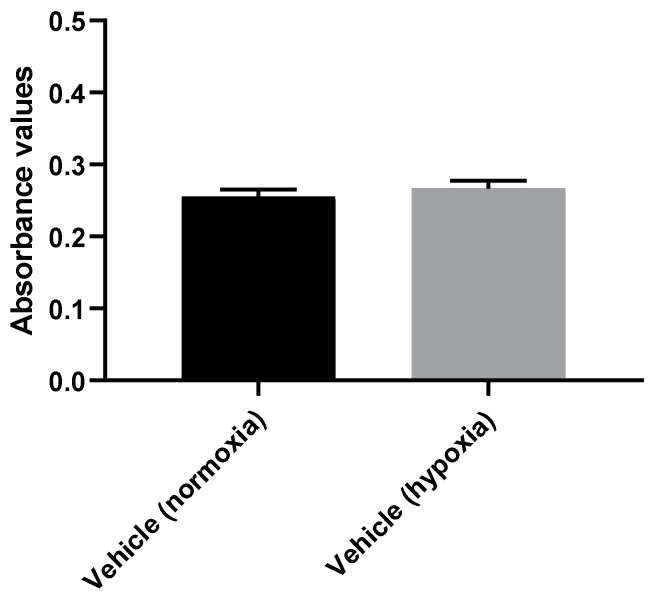
Comparative effect of induced hypoxia (with the hypoxia incubator) vs. normoxia on SH-SY5Y cell lines, 48 h. The results represent the mean ± SEM of three independent experiments.

**Figure 2:**
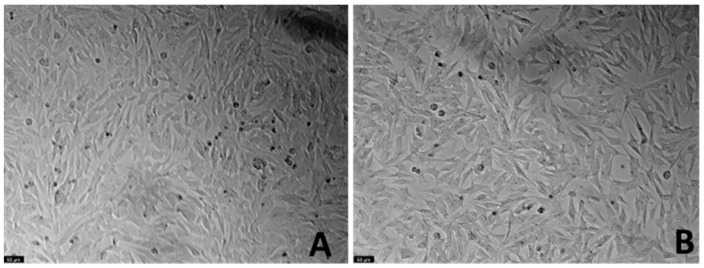
Representative images (100 × total magnification) of SH-SY5Y cells under (**A**) hypoxia and (**B**) normoxia.

To evaluate the effect of a hypoxic environment (2% O_2_, 10% CO_2_, and 88% N_2_ atmosphere) on the SH-SY5Y cells, we first compared both vehicles under hypoxia or normoxia conditions (21% O_2_). For hypoxia, one plate was placed in the hypoxia incubator and the other plate was placed under normoxia conditions. After 48 h, cellular viability was evaluated with MTT assay (Figure 1). Cellular morphology assessment was also carried out (Figure 2). All the procedures are described in Section 2.

These results reveal that the exposure of cells to hypoxia for 48 h did not differ from normoxia conditions. Indeed, absorbance values of 0.26 ± 0.01 (for normoxia conditions) and 0.27 ± 0.01 (for hypoxia conditions) were obtained. Cellular morphology was also similar in both conditions, revealing morphologically normal cells.

### 3.3. The Effect of the Hypoxia Incubator Chamber on SH-SY5Y Cellular Viability and Morphology after Drug Application

**Figure 3:**
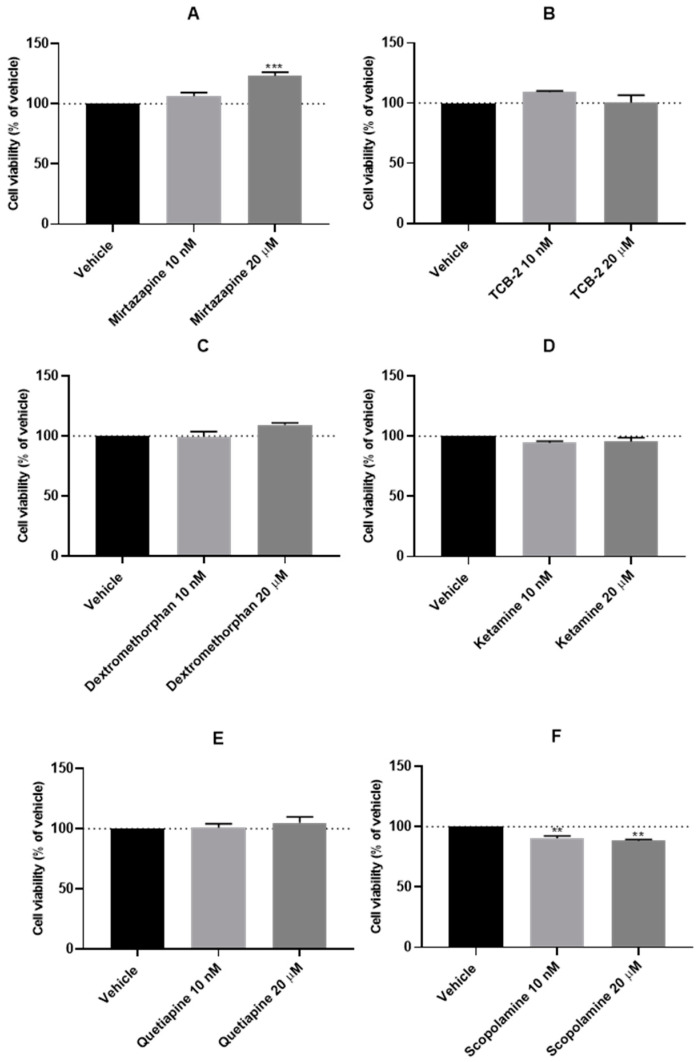

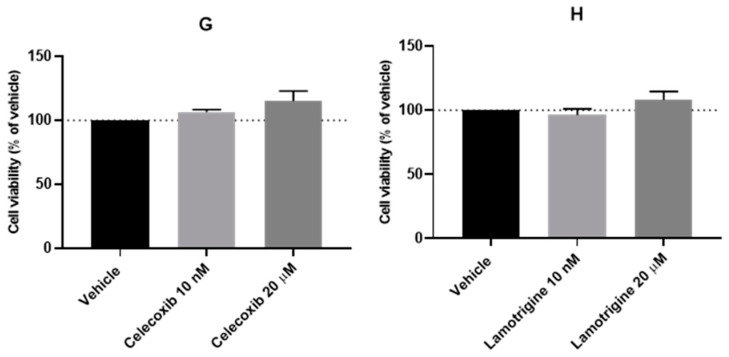
Effect of 48 h incubation of 10 nM and 20 μM of (**A**) Mirtazapine, (**B**) TCB-2, (**C**) Dextromethorphan, (**D**) Ketamine, (**E**) Quetiapine, (**F**) Scopolamine, (**G**) Celecoxib, and (**H**) Lamotrigine on the viability of SH-SY5Y cells, under hypoxia conditions, determined by MTT assay. The results represent the mean ± SEM of three independent experiments, expressed as the percentage of the hypoxia vehicle (100%). Statistically significant ** *p* < 0.01 and *** *p* < 0.001 vs. vehicle.

**Figure 4:**
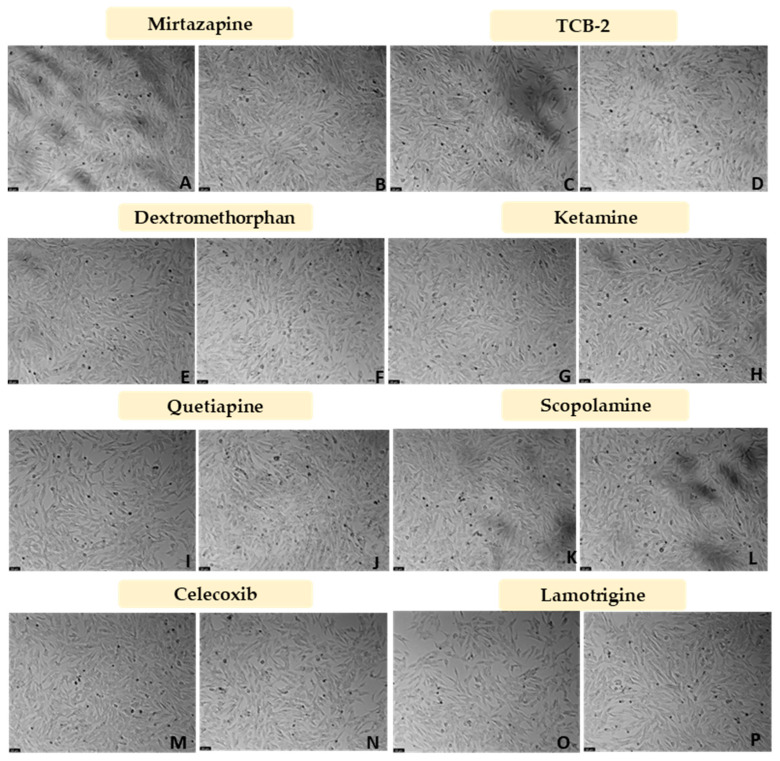
Representative images (100 × total magnification) of SH-SY5Y cells under hypoxia conditions. Cells were treated with (**A**) Mirtazapine 10 nM, (**B**) Mirtazapine 20 µM, (**C**) TCB-2 10 nM, (**D**) TCB-2 20 µM, (**E**) Dextromethorphan 10 nM, (**F**) Dextromethorphan 20 µM, (**G**) Ketamine 10 nM, (**H**) Ketamine 20 µM, (**I**) Quetiapine 10 nM (**J**) Quetiapine 20 µM, (**K**) Scopolamine 10 nM, (**L**) Scopolamine 20 µM, (**M**) Celecoxib 10 nM, (**N**) Celecoxib 20 µM, (**O**) Lamotrigine 10 nM, and (**P**) Lamotrigine 20 µM. The vehicle is represented in Figure 2A.

**Figure 5:**
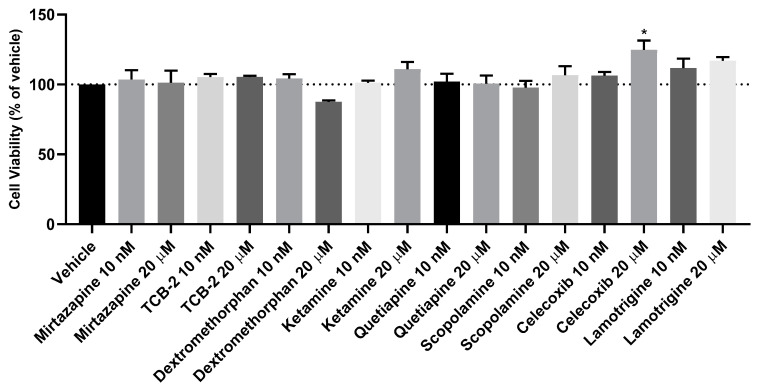
Effect of 48 h incubation of 10 nM and 20 μM of Mirtazapine, TCB-2, Dextromethorphan, Ketamine, Quetiapine, Scopolamine, Celecoxib, and Lamotrigine on the viability of SH-SY5Y cells, under normoxia conditions, determined by MTT assay, 48 h. The results represent the mean ± SEM of three independent experiments, expressed as the percentage of the vehicle (100%). Statistically significant * *p* < 0.05.

After the evaluation of the effect of a hypoxic environment on the SH-SY5Y cells, Mirtazapine (Figure 3A and Figure 4A,B), TCB-2 (Figure 3B and Figure 4C,D), Dextromethorphan (Figure 3C and Figure 4E,F), Ketamine (Figure 3D and Figure 4G,H), Quetiapine (Figure 3E and Figure 4I,J), Scopolamine (Figure 3F and Figure 4K,L), Celecoxib (Figure 3G and Figure 4M,N), and Lamotrigine (Figure 3H and Figure 4O,P) were added to these cells in concentrations of 10 nM and 20 μM, for 48 h, aiming to understand the effect of these drugs under a hypoxic environment. After that, cell viability was assessed using MTT (Figure 3), and morphological changes in the cells were also evaluated (Figure 4). Figure 5 demonstrates the effect of every single drug under normoxic conditions. All the procedures are described in Section 2.

Our results reveal that in general, all drugs led to similar values of cellular viability, identical to the values obtained with the vehicle. It is important to note that only Mirtazapine 20 µM increased cell viability under hypoxia (123.36 ± 2.98%). On the other hand, Nimodipine (20 µM) and Scopolamine (10 nM and 20 µM) decreased cell viability. Indeed, these drugs led to cell viability values of, respectively, 77.42 ± 2.24%, 90.47 ± 1.88%, and 88.35 ± 1.10%. These values were consistent with cellular morphology.

### 3.4. The Effect of Chemically Induced Hypoxia with Cobalt Chloride on SH-SY5Y Cellular Viability and Morphology

**Figure 6:**
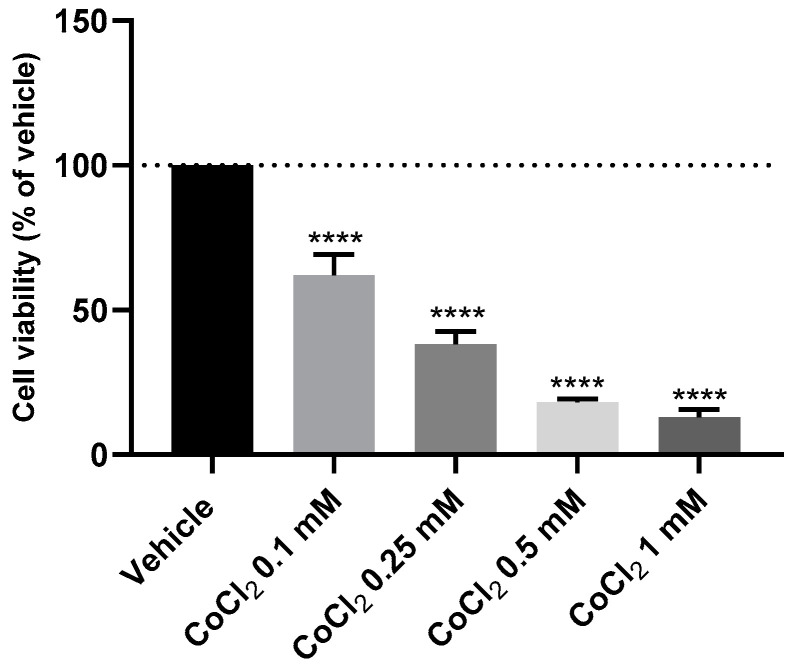
Effect of 48 h incubation of 0.1–1 mM of CoCl_2_, determined by MTT assay. The results represent the mean ± SEM of three independent experiments, expressed as the percentage of the vehicle (100%). Statistically significant **** *p* < 0.0001 vs. vehicle.

**Figure 7:**
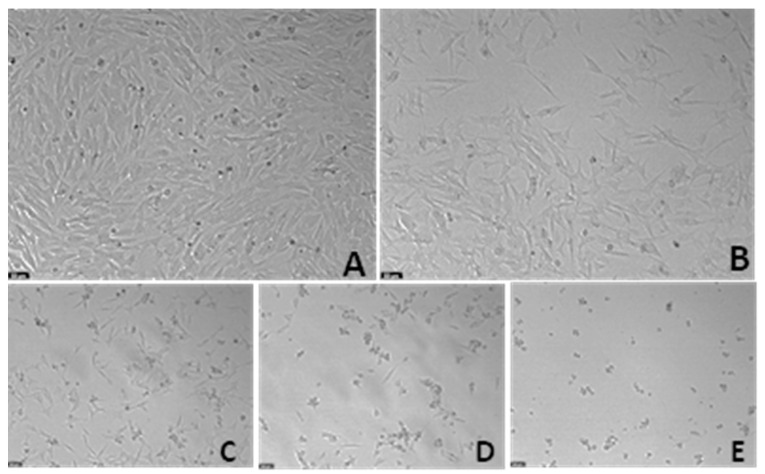
Representative images (100 × total magnification) of SH-SY5Y cells after 48 h exposure to (**A**) 1% sterilized water (vehicle), (**B**) CoCl_2_ 0.1 mM, (**C**) CoCl_2_ 0.25 mM, (**D**) CoCl_2_ 0.5 mM, and (**E**) CoCl_2_ 1 mM.

Aiming to choose the most suitable concentration of CoCl_2_ to chemically induce hypoxia, this compound was added to the cells for a period of 48 h, in crescent concentrations (0.1–1 mM), based on previous studies [24,25]. Cell viability values were obtained by MTT assay (Figure 6), and cellular morphology is represented in Figure 7. All the procedures are described in the Section 2.

These results demonstrate that CoCl_2_ decreased cellular viability in a concentration-dependent manner. Indeed, Figure 7 clearly shows that higher concentrations of this compound lead to fewer and more damaged cells, evidenced by a more rounded/small morphology. According to previous studies [24], we selected the concentration of CoCl_2_ that led to near half damage of the cells, ensuring that the damage response induced by hypoxia could be observed without damaging all the cells. Given this, we chose CoCl_2_ 0.1 mM (62.11 ± 3.17%) to induce hypoxia in the cells.

### 3.5. The Effect of Chemically Induced Hypoxia with Cobalt Chloride on SH-SY5Y Cellular Viability and Morphology after Drug Application

**Figure 8:**
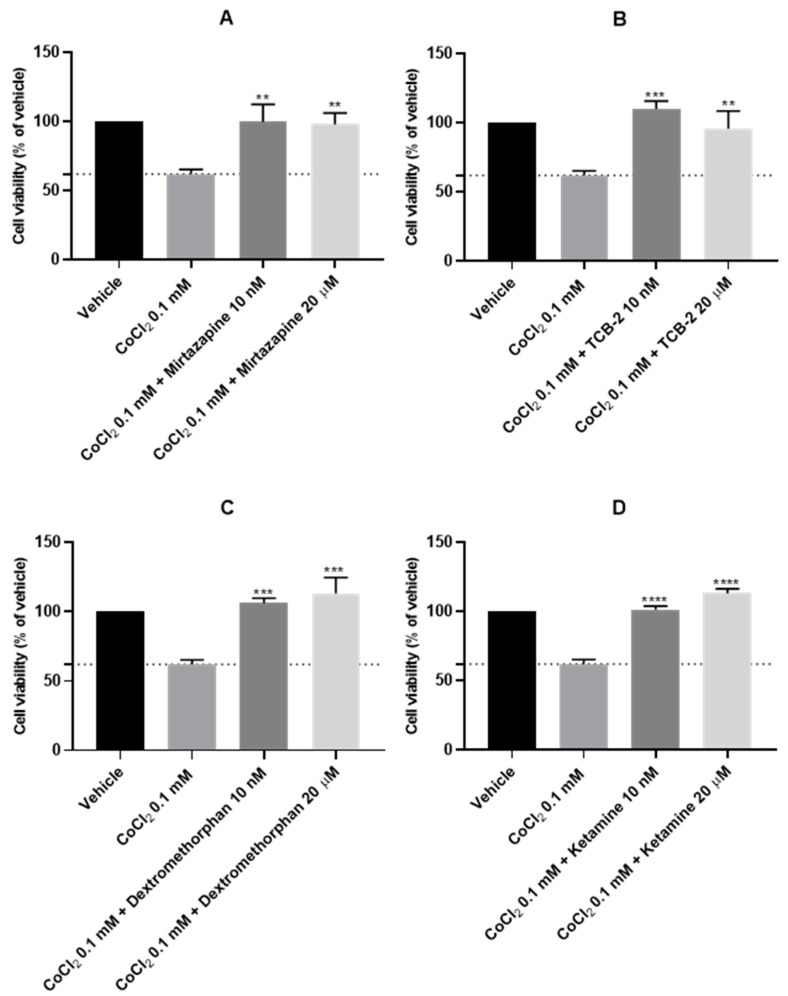

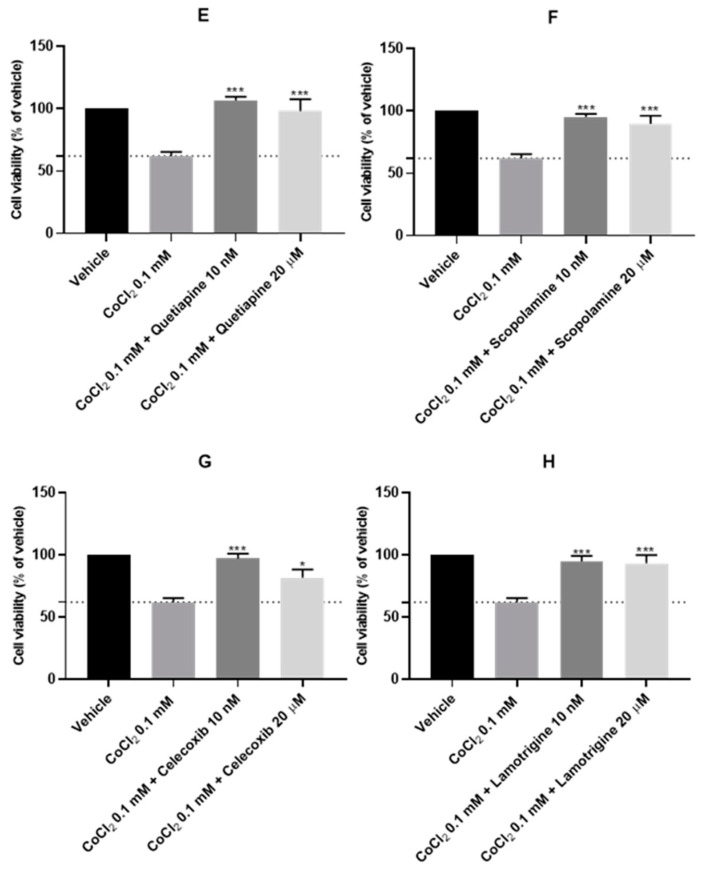
Effect of 48 h incubation of CoCl_2_ 0.1 mM in combination with 10 nM and 20 μM of (**A**) Mirtazapine, (**B**) TCB-2, (**C**) Dextromethorphan, (**D**) Ketamine, (**E**) Quetiapine, (**F**) Scopolamine, (**G**) Celecoxib, and (**H**) Lamotrigine on the viability of SH-SY5Y cells, determined by MTT assay. The results represent the mean ± SEM of three independent experiments, expressed as the percentage of the vehicle (100%). Statistically significant * *p* < 0.05, ** *p* < 0.01, *** *p* < 0.001, and **** *p* < 0.0001 vs. vehicle.

**Figure 9:**
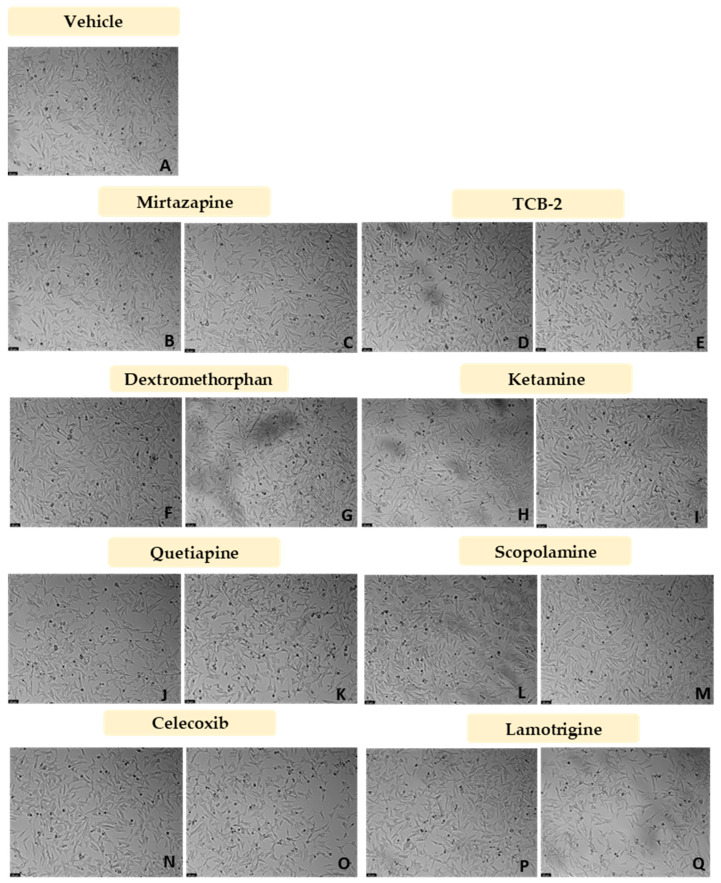
Representative images (100 × total magnification) of SH-SY5Y cells treated with (**A**) 0.2%DMSO/2% sterilized water (vehicle), (**B**) CoCl_2_ 0.1 mM + Mirtazapine 10 nM, (**C**) CoCl_2_ 0.1 mM + Mirtazapine 20 µM, (**D**) CoCl_2_ 0.1 mM + TCB-2 10 nM, (**E**) CoCl_2_ 0.1 mM + TCB-2 20 µM, (**F**) CoCl_2_ 0.1 mM + Dextromethorphan 10 nM, (**G**) CoCl_2_ 0.1 mM + Dextromethorphan 20 µM, (**H**) CoCl_2_ 0.1 mM + Ketamine 10 nM (**I**) CoCl_2_ 0.1 mM + Ketamine 20 µM, (**J**) CoCl_2_ 0.1 mM + Quetiapine 10 nM, (**K**) CoCl_2_ 0.1 mM + Quetiapine 20 µM, (**L**) CoCl_2_ 0.1 mM + Scopolamine 10 nM, (**M**) CoCl_2_ 0.1 mM + Scopolamine 20 µM, (**N**) CoCl_2_ 0.1 mM + Celecoxib 10 nM, (**O**) CoCl_2_ 0.1 mM + Celecoxib 20 µM, (**P**) CoCl_2_ 0.1 mM + Lamotrigine 10 nM, and (**Q**) CoCl_2_ 0.1 mM + Lamotrigine 20 µM.

After the evaluation of the most suitable concentration of CoCl_2_ to chemically induce hypoxia in the SH-SY5Y cells, CoCl_2_ 0.1 mM was combined with 10 nM and 20 μM of Mirtazapine (Figure 8A and Figure 9B,C), TCB-2 (Figure 8B and Figure 9D,E), Dextromethorphan (Figure 8C and Figure 9F,G), Ketamine (Figure 8D and Figure 9H,I), Quetiapine (Figure 8E and Figure 9J,K), Scopolamine (Figure 8F and Figure 9L,M), Celecoxib (Figure 8G and Figure 9N,O), and Lamotrigine (Figure 8H and Figure 9P,Q), aiming to understand the effect of these drugs on cells in chemically induced hypoxia, for 48 h. Cell viability was assessed using MTT (Figure 8), and morphological changes in the cells were also evaluated (Figure 9). All the procedures are described in Section 2.

Our results reveal that all the drugs, at both concentrations, increased cell viability values, compared to the cells treated with CoCl_2_ only. Indeed, Figure 9 clearly shows a higher number of cells after all the treatments, compared to CoCl_2_ only (Figure 7B).

### 3.6. The effect of Echinomycin and the Combination of Echinomycin with Cobalt Chloride on SH-SY5Y Cellular Viability and Morphology

**Figure 10:**
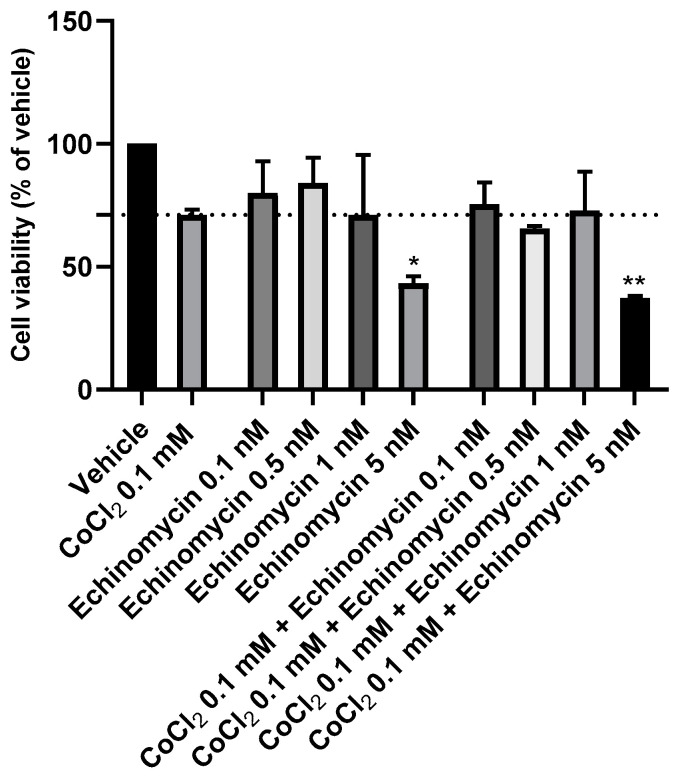
Effect of 48 h incubation of CoCl_2_ 0.1 mM, Echinomycin 0.1 nM-5 nM, and the combination of CoCl_2_ 0.1 mM with Echinomycin 0.1 nM–5 nM on the viability of SH-SY5Y cells, determined by MTT assay. The results represent the mean ± SEM of three independent experiments, expressed as the percentage of the vehicle (100%). Statistically significant * *p* < 0.05 and ** *p* < 0.01 vs. vehicle.

**Figure 11:**
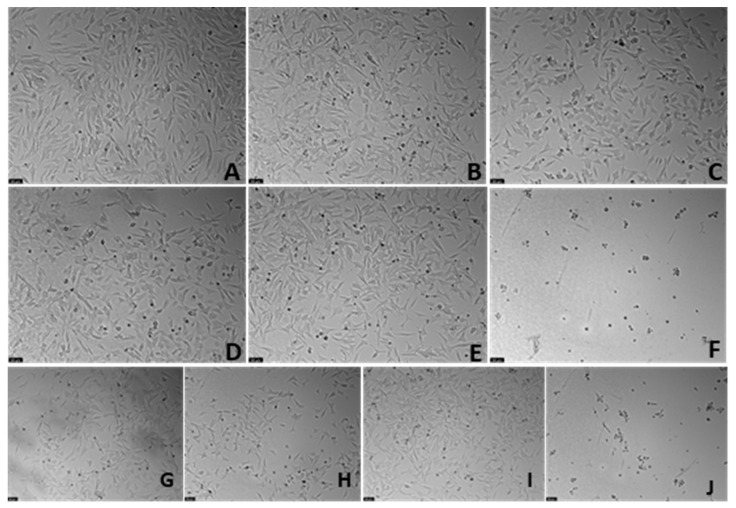
Representative images (100 × total magnification) of SH-SY5Y cells treated with (**A**) 0.2%DMSO/2% sterilized water (vehicle), (**B**) CoCl_2_ 0.1 mM, (**C**) Echinomycin 0.1 nM, (**D**) Echinomycin 0.5 nM, (**E**) Echinomycin 1 nM, (**F**) Echinomycin 5 nM, (**G**) CoCl_2_ 0.1 mM + Echinomycin 0.1 nM, (**H**) CoCl_2_ 0.1 mM + Echinomycin 0.5 nM (**I**) CoCl_2_ 0.1 mM + Echinomycin 1 nM, and (**J**) CoCl_2_ 0.1 mM + Echinomycin 5 nM.

Aiming to select the most suitable concentration of Echinomycin to inhibit HIF-1 DNA-binding and transcriptional activity, this compound was added to the cells for a period of 48 h, in crescent concentrations (0.1–5 nM), based on previous studies [26,27]. It is important to note that for this study, Echinomycin is used as a selective inhibitor of the HIF-1 protein and, thus, this drug should not induce high levels of cell death under normoxia. Cell viability values were obtained by MTT assay (Figure 10), and cellular morphology is represented in Figure 11. All the procedures are described in the Section 2.

Analyzing our results, it was evident that Echinonymcin decreased cellular viability in a concentration-dependent manner. Figure 11 clearly shows that higher concentrations of this compound lead to fewer and more damaged cells, evidenced by a more rounded/small morphology. Based on this data, we selected the concentration of 0.5 nM Echinomycin to proceed with the studies of inhibition of HIF-1 activity. Indeed, this concentration did not cause significant cell death under normoxic conditions. Additionally, when combined with CoCl_2_ 0.1 mM, this drug slightly decreases cell viability (5.48%), compared to CoCl_2_ alone.

### 3.7. The Effect of Echinomycin and the Combination of Echinomycin with Cobalt Chloride on SH-SY5Y Cellular Viability and Morphology after Drug Application

**Figure 12:**
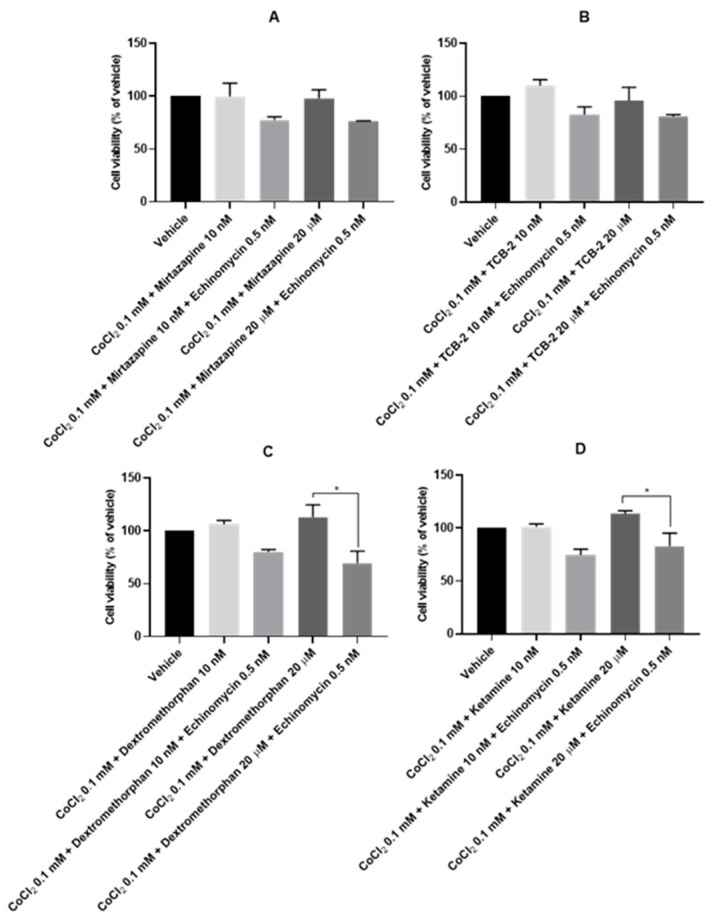

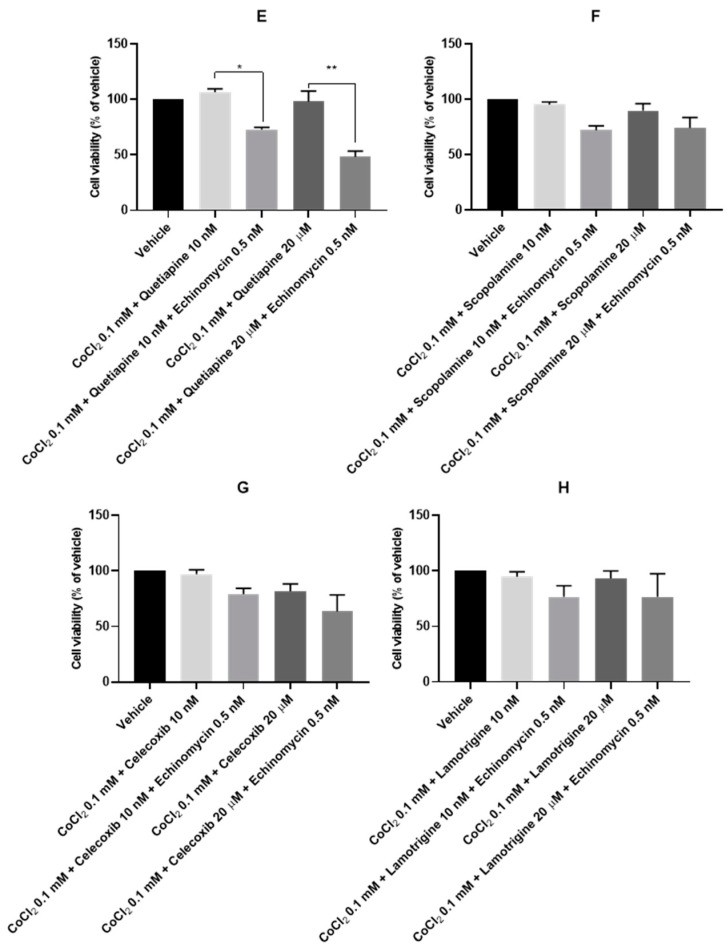
Effect of 48 h incubation of the combination of CoCl_2_ 0.1 mM, Echinomycin 0.5 nM and 10 nM/20 μM of (**A**) Mirtazapine, (**B**) TCB-2, (**C**) Dextromethorphan, (**D**) Ketamine, (**E**) Quetiapine, (**F**) Scopolamine, (**G**) Celecoxib, and (**H**) Lamotrigine on the viability of SH-SY5Y cells, determined by MTT assay. The results represent the mean ± SEM of three independent experiments, expressed as the percentage of the vehicle (100%). Statistically significant * *p* < 0.05 and ** *p* < 0.01.

**Figure 13:**
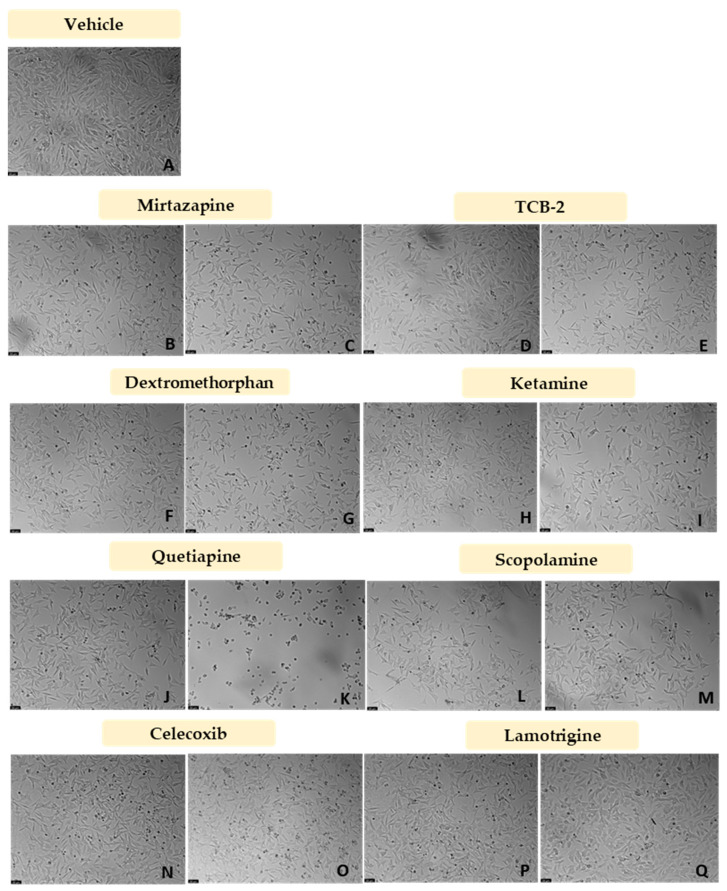
Representative images (100 × total magnification) of SH-SY5Y cells treated with (**A**) 0.3%DMSO/3% sterilized water (vehicle), (**B**) Echinomycin 0.5 nM + CoCl_2_ 0.1 mM + Mirtazapine 10 nM, (**C**) Echinomycin 0.5 nM + CoCl_2_ 0.1 mM + Mirtazapine 20 µM, (**D**) Echinomycin 0.5 nM + CoCl_2_ 0.1 mM + TCB-2 10 nM, (**E**) Echinomycin 0.5 nM + CoCl_2_ 0.1 mM + TCB-2 20 µM, (**F**) Echinomycin 0.5 nM + CoCl_2_ 0.1 mM + Dextromethorphan 10 nM, (**G**) Echinomycin 0.5 nM + CoCl_2_ 0.1 mM + Dextromethorphan 20 µM, (**H**) Echinomycin 0.5 nM + CoCl_2_ 0.1 mM + Ketamine 10 nM, (**I**) Echinomycin 0.5 nM + CoCl_2_ 0.1 mM + Ketamine 20 µM, (**J**) Echinomycin 0.5 nM + CoCl_2_ 0.1 mM + Quetiapine 10 nM, (**K**) Echinomycin 0.5 nM + CoCl_2_ 0.1 mM + Quetiapine 20 µM, (**L**) Echinomycin 0.5 nM + CoCl_2_ 0.1 mM + Scopolamine 10 nM, (**M**) Echinomycin 0.5 nM + CoCl_2_ 0.1 mM + Scopolamine 20 µM, (**N**) Echinomycin 0.5 nM + CoCl_2_ 0.1 mM + Celecoxib 10 nM, (**O**) Echinomycin 0.5 nM + CoCl_2_ 0.1 mM + Celecoxib 20 µM, (**P**) Echinomycin 0.5 nM + CoCl_2_ 0.1 mM + Lamotrigine 10 nM, and (**Q**) Echinomycin 0.5 nM + CoCl_2_ 0.1 mM + Lamotrigine 20 µM.

After the evaluation of the most suitable concentration of Echinomycin to inhibit the activity of HIF-1, Echynomicin 0.5 nM was combined with CoCl_2_ 0.1 mM and to 10 nM and 20 μM of Mirtazapine (Figure 12A and Figure 13B,C), TCB-2 (Figure 12B and Figure 13D,E), Dextromethorphan (Figure 12C and Figure 13F,G), Ketamine (Figure 12D and Figure 13H,I), Quetiapine (Figure 12E and Figure 13J,K), Scopolamine (Figure 12F and Figure 13L,M), Celecoxib (Figure 12G and Figure 13N,O), and Lamotrigine (Figure 12H and Figure 13P,Q), aiming to understand the effect of these drugs on cells in a chemically induced hypoxic environment with the inhibited activity of the adaptational response induced by HIF-1 protein in a hypoxic environment, as explained in the Section 1. Cell viability was assessed using MTT (Figure 12), and morphological changes in the cells were also evaluated (Figure 13). All the procedures are described in the Section 2.

To analyze these results, we compared the values of cellular viability obtained with the combination of CoCl_2_ and repurposed drugs with the values of cell viability obtained with the introduction of Echinomycin in these combinations. Our results revealed that all the drugs, at all the concentrations tested when combined with Echinomycin under hypoxia induced by CoCl_2_, decreased cell viability. These values were significant with Dextromethorphan 20 µM (69.45 ± 11.37%), Ketamine 20 µM (82.81 ± 12.42%), Quetiapine 10 nM (72.89 ± 1.93%) and 20 µM (48.21 ± 5.28%), and Nimodipine 20 µM (46.28 ± 5.39%). It is important to note the consistency between the MTT results and morphological evaluation of the cells. Indeed, in general, cells presented more rounded/small morphology after the introduction of Echinomycin to the drug combinations with CoCl_2_, revealing higher levels of cell damage.

## 4. Discussion and Conclusions

Several pieces of evidence highlight a connection between hypoxia and mood disorders [5,6,7,8]. Indeed, drugs that increase the expression of HIF-1 may be a promise in the therapeutic treatment of depression [13,14,15]. In this study, we aimed to explore the connection between hypoxic environments and the effect of non-cytotoxic (Figure 5) low (10 nM) and high (20 µM) concentrations of Mirtazapine [23] and other drugs with the potential to be repurposed as antidepressant drugs in SH-SY5Y human neuroblastoma cells, a cell line widely used in the research of neuropsychiatric and neurological disorders and mechanisms [28]. In fact, multiple drugs that were originally developed for different medical conditions have demonstrated promise in the treatment of depression. For this reason, several drugs were screened for BBB permeability. Our predictions identified Mirtazapine, TCB-2, Dextromethorphan, Ketamine, Quetiapine, Scopolamine, Celecoxib, and Lamotrigine as drugs with a high ability to cross this membrane and, therefore, these compounds were included in our study.

To induce hypoxia in cells, we exposed the cells to a hypoxia incubator chamber enriched with a 2% O_2_, 10% CO_2_, and 88% N_2_ atmosphere, contrasting with the 21% O_2_ present in normoxic conditions [29]. Additionally, we exposed the cells to CoCl_2_, which is widely used to artificially induce hypoxia in the cells, defined as chemical hypoxia [30]. Indeed, as described in the Section 1, this methodology of hypoxia induction is widely used because information regarding the methodology is available in the literature, it is substantially cheaper, and it can circumvent the difficulties in the regulation of the composition of O_2_/CO_2_ in studies with the hypoxia chamber [1]. Additionally, by controlling the concentration of CoCl_2_ added to the cells, there is the possibility of manipulating the response of the cells in a desirable period of study. Indeed, in our study, using the SH-SY5Y human neuroblastoma cells, we observed that the induction of hypoxia with the hypoxia incubator chamber showed practically no differences compared to normoxia conditions (Figure 1, Figure 2, Figure 3, Figure 4 and Figure 5), contrary to what we can observe through the induction of hypoxia with CoCl_2_, where lower cellular viability values were observed, comparing to a normoxic environment. In fact, the use of CoCl_2_ allowed us to observe a cellular response to the hypoxia stimulus for 48 h, which is why we continued this study with this model of chemical induction of hypoxia. After testing the drugs in a hypoxic environment induced by CoCl_2_, it was found that all these drugs increased cell viability (Figure 7 and Figure 8). Several mechanisms may explain these results. Indeed, the relationship between hypoxia and high levels of oxidative stress is known [31]. Previously, we studied the role of Mirtazapine in the production of oxygen free radicals, realizing that this drug tends to reduce oxidative stress [32], a mechanism that can also explain the responses observed with Mirtazapine in the context of hypoxia. However, a mechanism that could be behind these observed responses could be the increase in HIF-1 expression, important in cell proliferation/survival in hypoxic environments [12]. Thus, to understand whether the drugs were inducing this factor, we inhibited HIF-1 with Echinomycin, a potent HIF-1 inhibitor that works by inhibiting its DNA-binding and transcriptional activity [20]. Interestingly, our data revealed that all drugs decreased in cell viability when combined with Echinomycin (Figure 12 and Figure 13). Thus, we hypothesized that the increase in HIF-1 expression is possibly the mechanism behind the increase in cellular viability caused by the drugs in a hypoxic environment, despite the need for additional studies to confirm this hypothesis. Indeed, in particular, some studies state that Celecoxib [33] down-regulate HIF-1 expression. However, it is important to note that these responses may depend on the cellular/animal system under study, as well as the dosages/concentration of the drugs. Nevertheless, all these drugs present potential antidepressant characteristics [17,18,19] by interacting with inflammatory responses, neuronal factors, oxidative stress levels, neurotransmitters levels, and related pathways and/or HIF-1 expression, as evidenced in this manuscript. Table 2 lists examples of targets of these drugs known to be involved in depression, such as serotonin receptors. Little is known about the role of HIF-1 in depression, being a controversial topic of studies [15,34]. This initial investigation promotes further research into the involvement of hypoxia and HIF-1 in depression. This research holds great significance and exhibits potential for advancing depression therapy. Molecular biology techniques would be valuable as a next step. Indeed, Western blot analysis of HIF-1α protein levels to confirm our hypothesis would be valuable, as well as to understand how the different levels of hypoxia correlate with depression. Also, testing in other cell lines, including primary cell lines, would be very interesting regarding the translatability of findings.

## Figures and Tables

**Table 1:** Physicochemical properties of Mirtazapine, TCB-2, Dextromethorphan, Ketamine, Quetiapine, Scopolamine, Celecoxib, and Lamotrigine.

Drug	Molecular Weight (g/mol)	Diffusion Coefficient (cm^2^/s×0^5^)	LogP	LogD	Solubility (mg/mL)	BBB Permeability
Mirtazapine	265.36	0.782	2.659	2.282	0.269	High
TCB-2	234.50	0.893	2.341	0.251	4.469	High
Dextromethorphan	271.41	0.760	3.806	2.280	0.088	High
Ketamine	237.73	0.849	2.602	2.552	0.148	High
Quetiapine	406.00	0.646	2.701	1.993	0.359	High
Scopolamine	301.00	0.771	1.372	1.152	3.557	High
Celecoxib	381.38	0.701	3.808	3.807	0.006	High
Lamotrigine	259.06	0.917	1.998	1.998	0.029	High

**Table 2:** Summary of the targets known to be involved in depression for the drugs studied in this work and connected with serotonin receptors.

Drug	Targets Connected to Depression	References
**Mirtazapine**	Adrenergic α2, 5-HT2A and 5-HT3 receptors	[16]
**TCB-2**	5-HT2A	[19]
**Dextromethorphan**	NMDA, Sigma 1 and AMPA receptors, SERT	[35,36]
**Ketamine**	AMPA, 5-HT2, 5-HT1B and D2 receptor, BDNF	[37,38,39]
**Quetiapine**	5-HT2A and 5-HT1A receptors	[40,41]
**Scopolamine**	5-HT3 and AMPA receptors	[42,19]
**Celecoxib**	IL-6 levels	[43]
**Lamotrigine**	5-HT3 receptor, Voltage-sensitive sodium channel	[19,44]

5-HT1A: Serotonin receptor type 1A; 5-HT1B: Serotonin receptor type 1B; 5-HT2A: Serotonin receptor type 2A; 5-HT3: Serotonin receptor type 3; NMDA: N-methyl-D-aspartic acid; AMPA: α-amino-3-hydroxy-5-methyl-4-isoxazolepropionic acid; SERT: Serotonin transporter; D2: Dopamine receptor type 2; BDNF: Brain-derived neurotrophic factor; and Il-6: Interleukin 6.

## Data Availability

Not applicable.
